# Optimization of DIC-Tripolium Ecofriendly Extraction Process: Recovery of Hesperidin from Orange Byproducts, Antioxidant and α-Amylase Inhibition of Extracts

**DOI:** 10.3390/antiox12071346

**Published:** 2023-06-27

**Authors:** Mariem Ben Abdallah, Morad Chadni, Nouha M’hiri, Fanny Brunissen, Nesrine Rokbeni, Irina Ioannou, Karim Allaf, Colette Besombes, Nourhene Boudhrioua

**Affiliations:** 1 Laboratoire de Physiopathologie, Alimentation et Biomolécules, LR17ES03, Institut Supérieur de Biotechnologie de Sidi Thabet, Université de la Manouba, BP-66, Ariana-Tunis 2020, Tunisia; benabdallah.mariem94@gmail.com (M.B.A.); mhiri_nouha@yahoo.fr (N.M.); rokbeninesrine@gmail.com (N.R.); 2 URD Agro-Biotechnologies Industrielle (ABI), CEBB, AgroParisTech, 51110 Pomacle, France; morad.chadni@agroparistech.fr (M.C.); fanny.brunissen@agroparistech.fr (F.B.);; 3 Laboratoire des Sciences de l’Ingénieur Pour l’Environnement, LaSIE-UMR-CNRS-7356, Faculté des Sciences et Technologies, Université de La Rochelle, Avenue Michel Crépeau, CEDEX 01, 17042 La Rochelle, France

**Keywords:** orange byproducts, hesperidin, DIC treatment, Tripolium extraction, antioxidant activities, antidiabetic activity

## Abstract

This study aimed to investigate the effect of an innovative ecofriendly process—instant controlled pressure drop technology, also known as “détente instantanée contrôlée” or DIC—coupled with Tripolium extraction (DIC-Tripolium), on the hesperidin recovery, and antioxidant and antidiabetic activities of orange byproduct extracts. A DIC pretreatment was applied to partially dried orange byproducts (~16% wet basis). A central composite rotatable design (CCRD), composed of 13 experimental trials (four factorial points, four-star points, and five repetitions for the central point), was followed by a Tripolium process consisting of successive intermittent extraction periods using ethanol/water solvent at 20 ± 1 °C, 5 kPa for 5 min and *m*/*v* ratio = 5 g/50 mL. The DIC pretreatment, coupled with the Tripolium process, increased the extractability of hesperidin (from 1.55- to 4.67-fold compared to untreated DIC orange byproducts). The radical scavenging activities of the extracts were also enhanced or preserved in different DIC–Tripolium extracts. The α-Amylase inhibition percentage varied between 55.6 ± 0.02 and 88.30 ± 0.01% according to DIC–Tripolium conditions. The multi-criteria optimized condition of DIC–Tripolium extraction, allowing for the maximization of the hesperidin content, radical scavenging activities, iron chelating activity, and α-amylase inhibition of extracts, corresponds to a DIC saturated steam pressure of 599.4 kPa and a DIC pretreatment time of 38 s.

## 1. Introduction

Citrus is among the main consumed fruits, with approximately 158 million tons of worldwide production in 2021. Oranges (*Citrus × sinensis* (L.) *Osbeck*) production reached ~75.6 million tons in 2021 [1]. Orange byproducts, consisting of a mixture of seeds, pulp, and peels, are among the main wastes of the juice industry. They constitute about 30–40% *w*/*w* of the total orange weight and approximately 17–22 million tons are generated annually worldwide [2,3]. These agri-food byproducts undergo microbial fermentation and cause environmental issues [4]. They are often air-dried, used as animal feed, ground up, burned, or dumped in landfills. These abundant orange byproducts are affordable phenolic and flavonoid sources [5]. Their extracts’ yield and biological activity depend on the extraction process and operating conditions [6,7]. Citrus phenolic compounds have anti-inflammatory, cardio-protective [8], pharmacological [9], antimicrobial [10], anticancer [11,12], and antidiabetic activities [13].

Moreover, it was recently demonstrated that orange byproducts extract rich in hesperidin could inhibit SARS-CoV-2. The tremendous binding affinity of hesperidin with the human receptor binding domain of the angiotensin-converting enzyme 2 (RBD-ACE2) could explain its role in the prophylaxis of COVID-19. It could restrain the pro-inflammatory over-reaction of the immune system [14]. Hesperidin is also a natural antioxidant and antimicrobial agent against pathogenic bacteria [15,16]. Researchers have used various extraction techniques to recover functional compounds from vegetable byproducts [17]. The conventional solvent extraction (CSE) [18], the intensified conventional methods (enzymatic, ultrasound [19], microwave-assisted extraction), or non-conventional methods [20] (supercritical fluid extraction, pressurized liquid extraction) [21,22,23], generally involve extreme operating conditions: high extraction temperature, long extraction time, and different solvent/water ratios. Instant controlled pressure drop technology, known by its French acronym, DIC (détente instantanée contrôlée), was proven, during the last 30 years, as an innovative process successfully applied in fruit and vegetable processing and biomolecule extractions. DIC technology used high saturated steam pressure and short treatment time, allowing the development of different techno-functional and biological properties in the resulted expanded biological matrices. It is a thermo-mechanical process based on instantaneous thermodynamics theory. It consists of a saturated steam pressure treatment (from 100 to 900 kPa) exerted on the product for less than 1 min, followed by an abrupt and controlled pressure-drop (superior to 500 kPa per second) to reach a final vacuum of absolute pressure varying from 10 to 5 kPa, significantly lower than the atmospheric pressure. In these conditions, the DIC process allows for the autovaporization of water from the product, a quick cooling, and an expanded and porous final matrix. DIC technology could be used as a pretreatment process, reducing the duration of further drying or extraction processes and preserving high-quality final products [24,25]. DIC technology requires less treatment time and less energy than conventional processes. It is currently applied at research and industrial levels in food processing in European, American, and Chinese markets. It is used as a new pre-drying solution that improves the extraction yield of biomolecules and the antioxidant activity of the extract. Indeed, DIC extraction conditions limit thermal degradation and guarantee high-quality extracts by combining a brief treatment period with a temperature decline while depressurizing toward a vacuum; samples are exposed for a brief time (less than 60 s) to saturated steam pressure (P < 1000 kPa) at a high temperature (140–180 °C). DIC, combined with the conventional extraction methods, enhances phenolic compounds recovery from orange byproducts [26]. 

Moreover, when combined with the ultrasound-assisted process, DIC raises the essential oil yield from orange byproducts [27]. Ben Abdallah et al. [28] showed that DIC pretreatment, combined with conventional solvent extraction (CSE), ultrasound extraction (UAE), or accelerated solvent extraction (ASE), allowed increasing hesperidin recovery from orange byproducts and enhanced antioxidant activities. In contrast, the antidiabetic activity of the extract decreased when DIC was coupled with ASE. This decrease was attributed to the severe extraction conditions applied during ASE (130 °C for 30 min). Combining DIC pretreatment with a low-temperature extraction method would be more favorable for preserving the biological activities of the extracts. 

It was also reported that DIC may serve as an extraction operation itself [26]. Indeed, applying different cycles of DIC on DIC-textured materials reduces the extraction time by some minutes, and the operation has been called the Tripolium intermittent extraction process. It consists of the application, to the product, of different short 75 s cycles of the (1) DIC process, (2) solvent soaking, and (3) solvent draining steps. Thus, the Tripolium–DIC extraction process is based on the combination of three mechanisms occurring within the three successive stages of each cycle: (1) the DIC step for auto-vaporization, texturing, and expulsing processes, with (2) the internal under-vacuum invading the solvent flow through the injection of the solvent just at the vacuum stage of the DIC cycle, and (3) the external under-steam pressure expulsing the solvent flow, allowing the full expulsion of the solvent in the extract by establishing a total steam pressure usually higher than 200 kPa [25]. Tripolium extraction has been achieved in several minutes, instead of some hours, when the operation is performed without the DIC pretreatment, and about one hour with DIC-textured materials without the Tripolium intermittent extraction process. It has been proven to be a low-cost operation compared to conventional methods. The Tripolium extraction process is currently applied at a laboratory scale [24,25].

The central composite rotatable design (CCRD), including the main extraction parameters as variables and using response surface methodology, is always used to determine the optimal extraction conditions corresponding to the highest yield of target biomolecules. Several studies have used experimental designs to maximize the phenolic content and antioxidant activities of vegetable extracts based on conventional extraction [18], ultrasound extraction [19], and pressurized liquid extraction [29]. Generally, a mono-criterion optimization of the extraction method is performed considering the yield of the target biomolecule. This study aimed to investigate, for the first time, the application of the DIC–Tripolium process, an ecofriendly and gentle process (eco-extraction at 20 °C for a short time at 5 kPa), to the recovery of hesperidin from orange byproducts and bioactive extracts. A CCRD of experiments was used. The optimal conditions corresponding to the highest target biomolecule recovery, antioxidant, and antidiabetic activities were determined using mono-criterion and multi-criteria optimizations.

## 2. Materials and Methods

### 2.1. Plant Material and Sample Preparation

Maltese half-blood oranges (*Citrus × sinensis* (L.) *Osbeck*) from the region of Nabeul (Tunisia) were used. Orange byproducts were composed of 80% peel and 20% pulp. The initial moisture content of the sample is 75 ± 1.50 g/100 g wet basis. The sample was pre-dried for 20 h in the dark in an oven at 60 °C [30] until reaching a moisture content of ~15.60 ± 0.50 g/100 g wet basis [6,28]. The dried byproduct was ground, vacuum-packed in plastic bags of 200 g each, and then stored at −20 °C until its use for DIC pretreatment and DIC–Tripolium extraction. 

### 2.2. DIC Pretreatment and DIC–Tripolium Extraction

DIC equipment is composed of (1) a high-pressure/-temperature processing vessel where the sample to treat is placed, (2) a vacuum system and water-ring pump, and (3) an instant opening pneumatic valve assuring an abrupt connection between the vacuum tank and the processing vessel [20]. DIC treatment consists of the 4 following steps: initial vacuum, injection of saturated dry steam during the thermal treatment time, abrupt pressure-drop towards the vacuum, and then release to atmospheric pressure. A CCRD (Table 1), was applied to pre-dried orange byproducts. Two DIC operating parameters were retained: DIC saturated steam pressure (P) and DIC pretreatment time (t). Thirteen experimental trials were conducted. The CCRD implies 4 factorial points, 4 -star points, and 5 repetitions for the central point. The experimental trials were followed by a Tripolium extraction step consisting of successive intermittent periods using ethanol/water solvent at 20 ± 1 °C, 5 kPa for 5 min (*m*/*v* ratio = 5 g/50 mL). The applied solvent was 80% ethanol/water. This ratio was retained after preliminary extraction assays using different ethanol/water ratios (0–100%). Tripolium intermittent extraction step was also performed for untreated DIC samples (control, *n* = 3).

### 2.3. Scanning Electron Microscopy (SEM) Analysis

The microstructures of DIC-pretreated orange byproduct sample and control (untreated sample) were observed using an environmental SEM with energy dispersive X-ray spectroscopy, EDX (SEM/EDX: Q250, Thermo Scientific™ Analytical SEM, Karlsdorf-Neuthard Deutschland, Germany). The SEM combines a complete tungsten SEM with a powerful EDS detector for elemental analysis. The samples were placed on a covered support using carbon adhesive and were scanned in a partial vacuum (70 Pa) with an acceleration tension of 25 kV.

### 2.4. Analysis of Orange Byproduct Extracts 

#### 2.4.1. Analysis of Hesperidin by Ultra-High Performance Liquid Chromatography (UHPLC) 

A UHPLC analytical system (Thermo SCIENTIFIC Dionex UltiMate 3000, Wilmington, DC, USA), equipped with a quadratic pump, an auto-sampler, a column furnace, and a diode array detector, was used for the quantitative analysis of hesperidin content in different extracts, as described by Ben Abdallah et al. [28]. Before UHPLC injection, extracts were filtered through Millipore paper (0.22 µm). The injection volume was 5 µL and a constant flow rate of 0.8 mL/min was fixed. A gradient elution using acetonitrile solvent and formic acid 0.1% (*v*/*v*) in water was applied (Table 2) on a C18 column (ThermoScientific™Accucore™aQ, 100 × 3 mm, 2.6 μm particle size) maintained at 48 °C. The chromatograms were acquired at 285 nm and analyzed using Chromeleon software (version 6.8). Hesperidin was identified by comparing its relative retention time with the corresponding standard. 

The improvement yield of hesperidin recovery using DIC pretreatment was calculated according to the following equation [27]:

(1) $$\mathrm{HY}\;(\%)=\frac{{Hesperidin}_{DIC}-{Hesperidin}_{C}}{{Hesperidin}_{C}}\times 100$$

where HY (%) is the improvement yield of hesperidin recovery, ${Hesperidin}_{DIC}$ is the content of hesperidin determined in DIC-treated samples, and ${Hesperidin}_{C}$ is the content of hesperidin determined in untreated DIC samples (control).

#### 2.4.2. Determination of Radical Scavenging Activities Using DPPH Assay

The 2,2-diphenyl-1-picrylhydrazyl radical scavenging activity (DPPH-RSA) of the extracts was determined [6,28]. A 63 µM of DPPH was prepared by diluting 2.5 mg of DPPH in 100 mL of methanol. A volume of 400 µL of the sample extract was added to 2.4 mL of DPPH solution and the mixture was incubated in the dark for 30 min at room temperature. The reduction in DPPH radical was determined by measuring the absorbance at 515 nm. A concentration range (0–50 mg/L) of Trolox (6-hydroxy-2,5,7,8-tetramethylchroman-2-carboxylic acid) was used to perform the corresponding calibration curve, giving absorbance versus Trolox equivalents, TE (R^2^ = 0.99). TE (mg/L) was calculated from the calibration curve and used to determinate DPPH—RSA, which was expressed as mg Trolox equivalents per g of dry matter (mg TE/g DM) according to the following equation:

(2) $$DPPH-RSA(mg\;TE/g\;DM)=\frac{d\times TE\times v}{m_{DM}}$$

where d is the dilution, v is the total volume of the extract, and $m_{DM}$ is the mass of the sample based on dry mater.

#### 2.4.3. Determination of Radical Scavenging Activity Using ABTS Assay

The 2,2-azino-bis(3-ethylbenzothiazoline-6-sulfonic acid) radical scavenging activity (ABTS—RSA) of the extracts was evaluated as described by M’hiri et al. [6] and Ben Abdallah et al. [28]. The ABTS^•+^ radical cation was prepared by mixing an equal volume of a 7 mM ABTS diammonium salt aqueous solution with a 3 mM potassium persulfate (K_2_S_2_O_8_) solution. The mixture was then stored in the dark at room temperature for 12 h. To determine the ABTS—RSA, 50 μL of the diluted extract was mixed with 2 mL of ABTS reagent solution and the mixture was incubated at room temperature for 30 min. The absorbance was measured at 734 nm. A concentration range (0–250 mg/L) of Trolox was used to perform the corresponding calibration curve, giving absorbance versus TE (R^2^ = 0.99). TE (mg/L) was calculated from the calibration curve and was used to determinate ABTS—RSA expressed as mg TE/g DM according to Equation (2).

#### 2.4.4. Iron Chelating Activity (ICA)

The ICA of the extracts was assessed using the method described by Dinis et al. [31] and Ben Abdallah et al. [28]. Amounts of 50 μL of FeSO_4_ 7H_2_O (2 mM) and 500 μL of the orange byproduct extract were mixed. Then, 200 μL of 5 mM ferrozine (3-(2-pyridyl)-5,6-diphenyl-1,2,4-triazine-4′,4″-disulfonic acid sodium salt, C_20_H_13_N_4_NaO_6_S_2_) was added. The sample was then incubated for 15 min at 25 °C. The absorbance was measured at 562 nm. The concentration range (0–90 mg/L) of ethylenediaminetetraacetic acid (EDTA, C_10_H_16_N_2_O_8_) was used to perform the corresponding calibration curve, giving absorbance versus EDTA equivalents, EDTAE (R^2^ = 0.98). EDTAE (mg/L) was calculated from the calibration curve and was used to determine ICA expressed as mg EDTAE/g DM in a similar way to the previous antioxidant assays. 

#### 2.4.5. α-Amylase Inhibition Assay

The antidiabetic activity of orange byproduct extracts was assessed using α-amylase inhibition assay. Chromogenic 3.5-dinitrosalicylic acid (DNS) method, described by Benayad et al. [32] and Ben Abdallah et al. [28], was used. A volume of 500 µL of α-amylase was added to the extracts. The samples were then pre-incubated for 15 min at 37 °C in a shaker incubator (Thermo Scientific^TM^, Illkirch-Graffenstaden, France). Starch substrate solution (1%) was added to the samples. The mixtures were then incubated for 30 min in a boiling water bath. An amount of 1 mL DNS was added to stop the reaction. The mixtures were heated for 15 min in a boiling water bath and then cooled in an ice bath. The absorbance was measured at 540 nm. The α-amylase inhibition percentage, IP, was determined for all samples as follows:

(3) $$IP\left(\%\right)=\frac{{Abs}_{s}-{Abs}_{0}}{{Abs}_{s}}\times 100$$

where ${Abs}_{s}$ and ${Abs}_{0}$ are the absorbance of the sample and the absorbance of the control (blank), respectively.

### 2.5. Experimental Design and Statistical Analysis 

All experimental analyses were performed in triplicate on each sample of the CCRD and control samples. Statistical analysis of the CCRD was carried out using the software package STATGRAPHICS Centurion 19. A second-order empirical polynomial model was used to express the responses as a function of independent variables. Analysis of variance (ANOVA) was performed at a significance level of 5%. Response surface plots of the measured parameters (hesperidin content, ABTS—RSA, DPPH—RSA, ICA, and IP) were established. The mono-criterion optimization and multi-criteria optimization were performed using the desirability function available in the STATGRAPHICS Centurion 19 software (The Plains Virginia, VA, USA). For each measured response, the second-order regression equation was determined as follows:

(4) $$Y_{i}=\beta_{0}+\beta_{1}P+\beta_{2}t+\beta_{12}P\times t+\beta_{11}P^{2}+\beta_{22}t^{2}+ɛ$$

where Y_i_ is the predicted dependent response, β_j_ are the model coefficients, t and P, are the DIC pretreatment time (s) and DIC saturated steam pressure (kPa), respectively, and ɛ is the residue. After a first assessment of different model coefficients and ANOVA analysis, the model coefficients were then re-calculated using only the significant variables (*p*-value ≤ 0.05).

## 3. Results and Discussion 

### 3.1. Effect of DIC Treatment on Tripolium Extraction Efficiency 

Table 3 shows the hesperidin content, and the antioxidant and antidiabetic activities determined in the DIC-treated Tripolium and DIC untreated Tripolium samples. The HY (%) was also calculated. It can be noticed that the DIC-pretreatment allowed an increase in HY from 6.25% to ~375% (P = 541 kPa and t = 40.6 s) and enhanced the antioxidant (except for ICA, which was more variable) and antidiabetic activities of the extracts.

**Figure 1:**
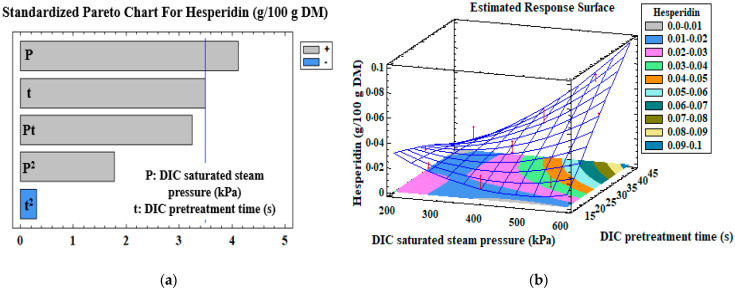
Standardized Pareto chart (**a**) and the corresponding estimated response surface (**b**) of hesperidin content (g/100 g DM). The red arrows correspond to the projection of experimental data of the CCRD.

**Figure 2:**
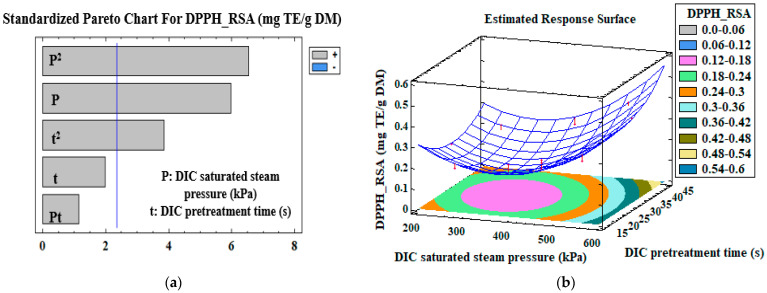

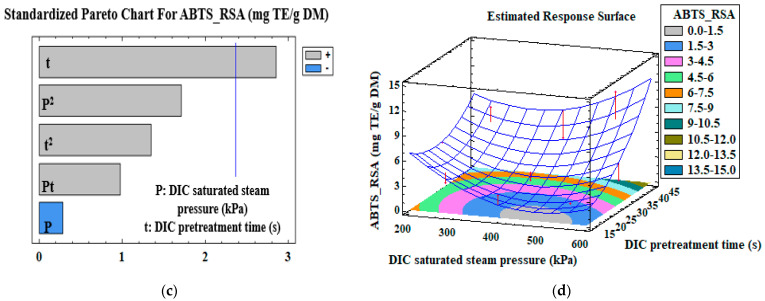
Standardized Pareto charts (**a**,**c**) and the corresponding estimated response surfaces (**b**,**d**) of the DPPH and ABTS radical scavenging activities (mg TE/g DM). The red arrows correspond to the projection of experimental data of the CCRD.

**Figure 3:**
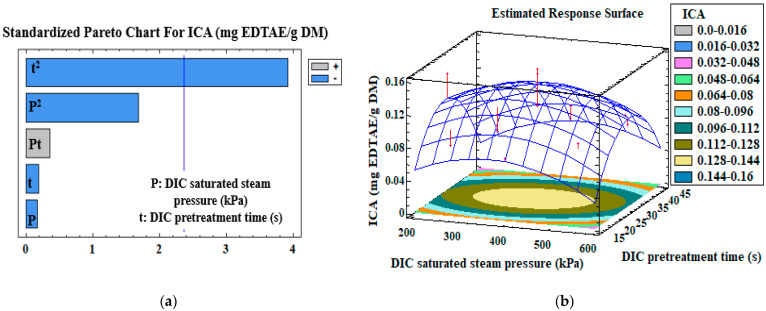
Standardized Pareto chart (**a**) and the corresponding estimated response surface (**b**) of iron chelating activity, ICA (mg EDTAE/g DM). The red arrows correspond to the projection of experimental data of the CCRD.

**Figure 4:**
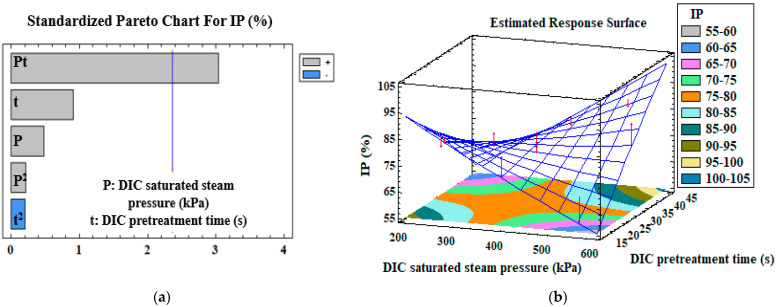
Standardized Pareto chart (**a**) and the corresponding estimated response surface (**b**) of α-amylase inhibition percentage, IP. The red arrows correspond to the projection of experimental data of the CCRD.

Pareto charts of the standardized effects of the DIC pretreatment (DIC saturated steam pressure, DIC pretreatment time, and their interaction), and the response surfaces of different measured variables, are presented in Figure 1, Figure 2, Figure 3 and Figure 4. The vertical line in the Pareto charts indicates the boundary between the major and minor impacts regarding the response depending on ANOVA. The size of each parameter represents the intensity of the estimated effect. The models were first assessed for the significance of their regression coefficients, and then evaluated using only the significant parameters (Table 4).

Figure 1a and Table 4 show that the DIC saturated steam pressure, (*p* = 0.004), DIC pretreatment time (*p* = 0.01), and their interaction, P × t (*p* = 0.01), are the main factors that positively affect hesperidin extractability (R^2^ = 0.80). The increase in DIC saturated steam pressure from 240 to 600 kPa, for a DIC pretreatment time of 30 s, induces an increase in hesperidin content from 0.017 ± 0.004, 0.027 ± 0.003 to 0.058 ± 0.003 g/100 g DM. For a DIC saturated steam pressure of 541 kPa, an increase in DIC treatment time from 19.4 to 40.6 s allowed an increase in hesperidin content from 0.01 ± 0.05 to 0.076 ± 0.001 g/100 g DM. Similarly, for a DIC saturated steam pressure of 400 kPa, an increase in DIC pretreatment time from 15 to 45 s led to an increase in hesperidin content from 0.019 ± 0.001 to 0.028 ± 0.07 g/100 g DM.

Based on the results of the Pareto charts and response surface plots, it is worth noticing that the increase in both DIC pretreatment time and saturated steam pressure resulted in an improvement in the radical scavenging activities (DPPH—RSA, ABTS—RSA) of the extracts and of hesperidin content. This result is attributed to the fact that the DIC saturated steam pressure and DIC pretreatment time increase the effective diffusivity of hesperidin, as Louati et al. [26] reported. Safdar et al. [33] reported that the hesperidin content recovered from orange byproducts using conventional solvent extraction (ethanol = 50%, time = 20 h, temperature = 40 °C) was 0.0092 g/100 g. This hesperidin content is two-fold lower than the untreated DIC–Tripolium control sample (0.016 ± 0.002 g/100 g, Table 3) and 8-fold lower than the maximum value reached in the DIC-treated Tripolium sample. Thus, the DIC–Tripolium process improved the hesperidin content compared to the conventional solvent extraction. Allaf et al. [34] reported that 60 min extraction at 40 °C, with ethanol 80%, of DIC-pretreated orange byproducts allows the recovery of 0.64 ± 2.7 × 10^−2^ g/100 g DM of hesperidin. This hesperidin content is almost 10-fold superior to the maximum hesperidin content determined in this study. This difference is attributed to the different DIC pretreatment time (120 s versus 41 s in this study) and the number of applied pretreatment DIC cycles (6 cycles versus 1 cycle) before the extraction step. Both the DIC pretreatment time and saturated steam pressure improved the yield of hesperidin recovery by approximately 375% compared to untreated samples. It was reported that DIC treatment intensified the explosion of the matrix and facilitated the release of phenolic compounds from the cell matrix. This abrupt temperature drop significantly impacts the vapor level issued from auto-vaporization, which may also generate cell wall rupture. This cell wall breakdown significantly enlarges the remaining pores, allowing polyphenols diffusion throughout the extraction process [35].

Similarly, an increase in the DIC pretreatment time leads to an increase in the solvent penetration rate, which contributes to favorable chemical solubility, increased solvent diffusivity, and reduced solvent viscosity, which improves the ability of the compound to penetrate matrix structures and boosts mass transfer rates, leading to high compound extractability [25,36]. Furthermore, the solvating property of ethanol and the extraction temperature influence the solubilization of amphiphilic molecules, such as hesperidin. We had to increase the saturated steam pressure, pretreatment time, and treatment cycles to improve the HY (%) from orange byproducts with DIC–Tripolium extraction. Figure 2a showed that the variation of the DPPH—RSA of the extracts was significantly (*p* = 0.0006 influenced (R^2^ = 0.88) by the DIC saturated steam pressure, the square of the DIC saturated steam pressure (*p* = 0.0003), and the square of the DIC pretreatment time (*p* = 0.009).

In addition, Figure 2b and Table 3 show that an increase in DIC saturated steam pressure from 200 to 600 kPa, for a DIC pretreatment time of 30 s, led to an increase in the DPPH—RSA from 0.210 ± 0.09 to 0.411 ± 0.01 mg TE/g DM. For a DIC saturated steam pressure of 541 kPa, the increase in the DIC pretreatment time from 19.4 to 40.6 s increased the DPPH—RSA from 0.266 ± 0.01 to 0.399 ± 0.07 mg TE/g DM. Similarly, at the same DIC pretreatment time and saturated steam pressure of 259 kPa, the DPPH—RSA activity increased from 0.179 ± 0.04 to 0.239 ± 0.002 mg TE/g DM. Furthermore, the DIC pretreatment time positively influenced (*p*-value = 0.02) the ABTS—RSA of the extracts but with a low model correlation coefficient (R^2^ = 0.40) (Figure 2c). At a constant DIC saturated steam pressure of 541 kPa, the increase in DIC pretreatment time from 19.4 to 40.6 s induced a significant rise in ABTS—RSA from 1.699 ± 0.003 to 11.460 ± 0.01 mg TE/g DM (Table 3 and Figure 2d). For a DIC saturated steam pressure of 259 kPa and a DIC pretreatment time varying from 15 to 45 s, ABTS—RSA increased from 2.833 ± 0.06 to 8.299 ± 0.01 mg TE/g DM. For a pretreatment time varying from 15 to 45 s and a constant saturated steam pressure of 400 kPa, ABTS—RSA increased from 2.760 ± 0.006 to 4.553 ± 0.03 mg TE/g DM. The ICA of the extract was negatively affected (R^2^ = 0.60) by the square of DIC pretreatment time (*p*-value = 0.002; Figure 3a). The ICA slightly increased from 0.0765 ± 0.0006 to 0.0862 ± 0.0006 mg EDTAE/g DM for a DIC pretreatment time varying from 15 to 45 s and a constant saturated steam pressure of 400 kPa. For DIC saturated steam pressure of 400 kPa and pretreatment time varying from 15 to 30 s, the ICA increased from 0.0765 ± 0.0006 to 0.140 ± 0.01 mg EDTAE/g DM. However, at a lower DIC saturated steam pressure of 259 kPa and a pretreatment time varying from 19.4 to 40.6 s, the ICA decreased from 0.0774 ± 0.0006 to 0.0559 ± 0.007 mg EDTAE/g DM (Table 3 and Figure 3b). DIC pretreatment improved the ABTS—RSA of extracts by approximately 441% (Table 3) for orange byproducts. The ABTS assay appears more appropriate and suitable for determining the capacity of citrus extracts to scavenge free radicals. The DPPH—RSA assay is selective because it does not react with aromatic acids or flavonoids that do not contain OH groups in the B-ring [37]. In general, ICA antioxidant activity gives lower activity in orange byproduct extracts than DPPH—RSA and ABTS—RSA. These results agree with those of Senol et al. [38]; the authors reported that the ICA in 80% ethanolic extract of citrus peel was 12%.

Figure 4a shows that the antidiabetic activity, expressed as IP, was positively correlated with the interaction DIC pretreatment time x DIC saturated steam pressure (*p* = 0.01), but the correlation coefficient of the model is low (R^2^ = 0.53). Figure 4b and Table 4 show that, for a DIC pretreatment time varying from 19.4 to 40.6 s and a saturated steam pressure of 541 kPa, the IP significantly rose from 55.6 ± 0.02 to 88.3 ± 0.01%.

The extraction method and the interaction between DIC pretreatment time and DIC saturated steam pressure affected the antidiabetic activity of orange byproducts. The longer the DIC saturated steam pressure and DIC pretreatment time, the higher the IP values. The endo-enzyme α-amylase catalyzes the hydrolysis of internal α-1,4-glycosidic linkages, and glucose is transported in portal circulation after being absorbed by the gut. Amylase inhibitors in the digestive system postpone the breakdown of polysaccharides, which slows glucose absorption and lowers blood glucose levels. It was reported that flavonoids and other phenolic compounds strongly inhibit amylase activity [13]. The highest percentage of inhibition determined in the DIC-treated Tripolium extract of orange byproducts (IP ~88%) found in this study was higher than the values reported in previous studies using conventional ethanol/water (78%) extraction [32].

### 3.2. Effect of DIC Treatment on Microstructure

**Figure 5:**
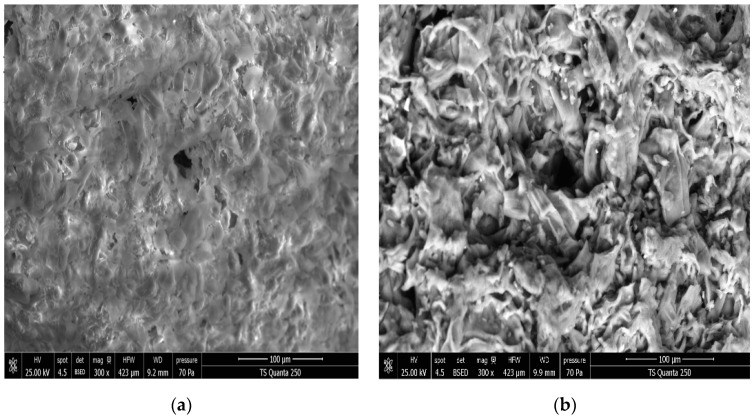
Scanning electron micrographs at 100 µm of untreated (**a**) and DIC-treated (**b**) orange byproducts at P = 400 kPa and t = 30 s. P: DIC saturated steam pressure (kPa), t: DIC pretreatment time (s).

**Figure 6:**
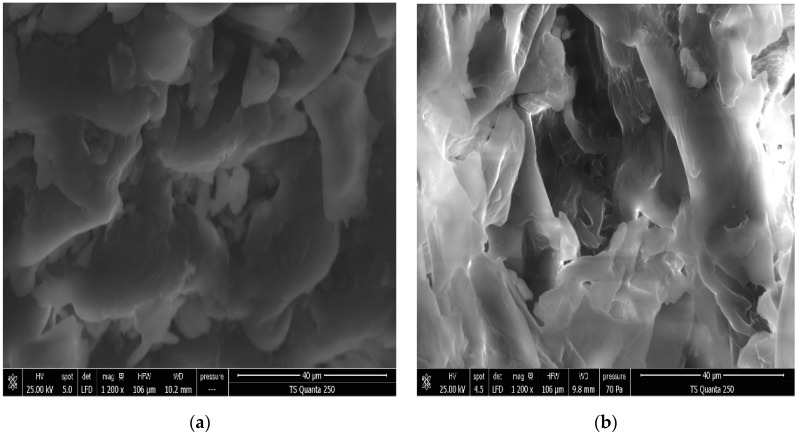
Scanning electron micrographs at 40 µm of untreated (**a**) and DIC-treated (**b**) orange byproducts at P = 400 kPa and t = 30 s. P: DIC saturated steam pressure (kPa), t: DIC pretreatment time (s).

The SEM micrographs (Figure 5 and Figure 6) showed a microstructural change in the sample after DIC treatment. Untreated DIC orange byproducts (Figure 5a and Figure 6a) exhibited a tight cell structure with a few tiny pores compared to the DIC-treated material (Figure 5b and Figure 6b). The DIC treatment led to a more expanded and porous structure. This agrees with the literature. Indeed, it is well known that DIC treatment can affect the product microstructure by generating a more porous structure and inducing a cell wall breakdown. This enhances cell permeability and increases biomolecule diffusivity into the solvent [24,26,27].

### 3.3. Mono-Criterion Optimization

Table 5 presents the optimal conditions for mono-criterion optimization (considering each response alone: maximized hesperidin content, DPPH—RSA, ABTS—RSA, ICA, and IP) of DIC–Tripolium extraction applied to orange byproducts.

It can be noticed that the optimal conditions for hesperidin recovery and DPPH—RSA are identical (DIC saturated steam pressure = 599.4 kPa and DIC pre-treatment time ~45 s), whereas a lower DIC saturated steam pressure and a shorter DIC pretreatment time are required for the optimal values of ICA and IP.

### 3.4. Multi-Criteria Optimization

Only the most crucial and significant extraction factors of different models were retained in the multi-criteria optimization of DIC–Tripolium extraction. Multi-criteria optimization was performed for hesperidin content, DPPH—RSA, ICA, and IP responses, for which the corresponding models’ correlation coefficients ranged from 0.53 to 0.88. The optimum conditions for DIC–Tripolium extraction from orange byproducts were: DIC pretreatment time = 38 s and DIC saturated steam pressure = 599.4 kPa. These optimum values represent the highest ones obtained in the range of variations in −α and +α levels and correspond to the predicted responses presented in Table 6. It can be noticed that the optimal value of DIC saturated steam pressure is identical to that obtained for hesperidin and DPPH—RSA in mono-criterion optimization. The predicted optimal values of hesperidin, DPPH—RSA, ICA, and IP (Table 6) were close to the highest experimental values evaluated in similar DIC–Tripolium process conditions.

## 4. Conclusions

Instant controlled pressure drop (détente instantanée contrôlée (DIC), coupled with Tripolium extraction (DIC–Tripolium), is suggested as an innovative ecofriendly extraction process (using a short extraction time of 5 min, a low extraction temperature ~20 °C, and an ecofriendly ethanol/water solvent) that increased hesperidin recovery and improved the antioxidant activities of orange byproduct extracts compared to the untreated DIC samples. DIC pretreatment time and saturated steam pressure significantly influenced the HY (%) from the orange byproducts and DPPH-radical scavenging activity of the extracts. DIC–Tripolium extraction, at a DIC saturated steam pressure of 599.4 kPa and a total DIC–Tripolium treatment time of ~6 min, allowed the maximization of the target molecule recovery and antioxidant and antidiabetic activities of the orange byproduct extracts. The SEM analysis showed that the DIC pretreatment generates a more expanded and porous material, improving extraction efficiency. Further investigations of the Tripolium extraction process, considering solvent recovery and process life cycle (process performance, sustainability, and investment cost), should be performed before its sustainable application at an industrial scale.

## Figures and Tables

**Table 1:** The central composite rotatable design, CCRD: independent variables of instant controlled pressure-drop (DIC)-pretreatment of orange byproducts and their ranges.

	DIC Saturated Steam Pressure (kPa)	DIC Pretreatment Time (s)
Point min (−α)	200	15
Point (−1):	259	19
Central point	400	30
Point (+1):	541	41
Point max (+α)	600	45

**Table 2:** Flow gradient used for ultra-high performance liquid chromatography (UHPLC) analysis of hesperidin.

Time (min)	Flow (mL/min)	Acetonitrile (%)	Formic Acid 0.1% (*v*/*v*) in Water (%)
0	0.8	2	98
3	0.8	14	86
5.5	0.8	20	80
9	0.8	50	50
9.5	0.8	50	50
10	0.8	95	5
11.5	0.8	95	5
12	0.8	2	98
14	0.8	2	98

**Table 3:** Comparison of hesperidin content, antioxidant, and antidiabetic activities of orange byproducts treated and untreated with instant controlled pressure drop, DIC, technology coupled with Tripolium extraction (DIC–Tripolium).

Trials	P (kPa)	T (°C)	t (s)	DPPH—RSA (mg TE/g DM)	ABTS—RSA (mg TE/g DM)	ICA (mg EDTAE/g DM)	IP (%)	Hesperidin (g/100 g DM)	HY (%)
Control (*n* = 3)	C	C	C	0.138 ± 0.004	2.594 ± 0.05	0.104 ± 0.01	82 ± 0.1	0.016 ± 0.002	0
Central point (*n* = 5)	400	143.67	30	0.151 ± 0.01	2.471 ± 0.02	0.140 ± 0.01	77 ± 0.1	0.027 ± 0.003	68.75
1	600	158.90	30	0.411 ± 0.01	2.914 ± 0.03	0.099 ± 0.0006	88.2 ± 0.1	0.058 ± 0.003	262.5
2	400	143.67	45	0.240 ± 0.01	4.553 ± 0.03	0.0862 ± 0.0006	76.50 ± 0.01	0.028 ± 0.07	75
3	541	154.92	40.6	0.399 ± 0.07	11.460 ± 0.01	0.0891 ± 0.004	88.3 ± 0.01	0.076 ± 0.003	375
4	541	154.92	19.4	0.266 ± 0.01	1.699 ± 0.003	0.0941 ± 0.0005	55.6 ± 0.02	0.01 ± 0,05	6.25
5	259	128.55	19.4	0.179 ± 0.04	2.833 ± 0.06	0.0774 ± 0.0006	81.90 ± 0.05	0.016 ± 0.008	0
6	259	128.55	40.6	0.239 ± 0.002	8.299 ± 0.01	0.0559 ± 0.0007	73.3 ± 0.1	0.025 ± 0.001	56.25
7	200	120.22	30	0.210 ± 0.09	5.607 ± 0.04	0.1380 ± 0.02	73.60 ± 0.05	0.017 ± 0.004	6.25
8	400	143.67	15	0.253 ± 0.01	2.760 ± 0.006	0.0765 ± 0.0006	81.1 ± 0.1	0.016 ± 0.002	0

P: DIC saturated steam pressure, T: temperature of the sample in DIC vessel, t: DIC pretreatment time, DPPH—RSA: 2,2-Diphenyl-1-picrylhydrazyl radical scavenging activity, ABTS—RSA: 2,2-azinobis-3-ethylbenzothiozoline-6-sulphonate radical scavenging activity, RSA: radical scavenging activity, TE: Trolox equivalent, ICA: iron chelating activity, EDTAE: ethylenediaminetetraacetic acid equivalent, IP: α-amylase inhibition percentage, HY (%): improvement yield of hesperidin recovery. The standard deviation corresponds to three replications of the analysis for trials 1–8: 3 replications of the Tripolium process and 3 replications of the analysis for the control sample, and 5 replications of the DIC–Tripolium process, and 3 replications of the analysis for the central point.

**Table 4:** Polynomial equation coefficients for each response variable of instant controlled pressure drop, DIC, technology coupled with Tripolium (DIC–Tripolium) extraction applied to orange byproducts.

Response Variable	Source	Significant Regression Coefficients		*p*-Value
Hesperidin (g/100 g DM)	Model	β_0_	7.680 × 10^−2^	
	P (kPa)	β_1_	−2 × 10^−4^	0.004
	t (s)	β_2_	−3 × 10^−3^	0.01
	P × t	β_12_	1 × 10^−5^	0.01
		R^2^	0.80	
DPPH—RSA (mg TE/g DM)	Model	β_0_	9.6 × 10^−1^	
	P (kPa)	β_1_	−2.7 × 10^−3^	0.0006
	P^2^	β_11_	4 × 10^−6^	0.0003
	t^2^	β_22_	4.1 × 10^−4^	0.009
		R^2^	0.88	
ABTS—RSA (mg TE/g DM)	Model	β_0_	−2.25	
	t (s)	β_2_	0.21	0.02
		R^2^	0.40	
ICA (mg EDTAE/g DM)	Model	β_0_	−0.13	
	t^2^	β_22_	−3 × 10^−4^	0.002
		R^2^	0.60	
IP (%)	Model	β_0_	160.1	
	P × t	β_12_	7 × 10^−3^	0.005
		R^2^	0.53	

P: DIC saturated steam pressure, t: DIC pretreatment time, DPPH—RSA: 2,2-Diphenyl-1-picrylhydrazyl radical scavenging activity, ABTS—RSA: 2,2-azinobis-3-ethylbenzothiozoline-6-sulphonate radical scavenging activity, RSA: radical scavenging activity, TE: Trolox equivalent, ICA: iron chelating activity, EDTAE: ethylenediaminetetraacetic acid equivalent, IP: α-amylase inhibition percentage.

**Table 5:** Predicted optimum values for mono-criterion optimization of instant controlled pressure drop, DIC technology coupled with Tripolium (DIC–Tripolium) extraction applied to orange byproducts.

Optimal Condition	Hesperidin (g/100 g DM)	DPPH—RSA (mg TE/g DM)	ABTS—RSA (mg TE/g DM)	ICA (mg EDTAE/g DM)	IP (%)
DIC saturated steam pressure (kPa)	599.4	599.4	400	400	200.6
DIC pretreatment time (s)	44.99	44.99	44.99	30	15.01
Predicted values	0.091	0.493	7.176	0.130	97.93

DPPH—RSA: 2,2-Diphenyl-1-picrylhydrazyl radical scavenging activity, ABTS—RSA: 2,2-azinobis-3-ethylbenzothiozoline-6-sulphonate radical scavenging activity, RSA: radical scavenging activity, TE: Trolox equivalent, ICA: iron chelating activity, EDTAE: ethylenediaminetetraacetic acid equivalent, IP: α-amylase inhibition percentage.

**Table 6:** Multi-criteria optimization for orange byproducts.

Response	Optimal Value
Hesperidin (g/100 g DM)	0.071
DPPH-RSA (mg TE/g DM)	0.427
ICA (mg EDTAE/g DM)	0.111
IP (%)	88.25

DPPH—RSA: 2,2-Diphenyl-1-picrylhydrazyl radical scavenging activity, RSA: radical scavenging activity, TE: Trolox equivalent, ICA: iron chelating activity, EDTAE: ethylenediaminetetraacetic acid equivalent, IP: α-amylase inhibition percentage.

## Data Availability

Data is contained within the article.
